# Normative body mass-adjusted reference ranges of magnetic resonance imaging signs commonly used in diagnosing idiopathic intracranial hypertension in a healthy standard population

**DOI:** 10.1038/s41598-024-54975-0

**Published:** 2024-02-24

**Authors:** Rike Kobrow, Stefan Gross, Robert Fleischmann, Jörg Baldauf, Sönke Langner, Sebastian Strauss

**Affiliations:** 1 Institute of Diagnostic Radiology and Neuroradiology, University Medicine Greifswald, Greifswald, Germany; 2 Department of Neurology, University Medicine Greifswald, Ferdinand-Sauerbruch-Str. 1, 17475 Greifswald, Germany; 3 DZHK (German Center for Cardiovascular Research), Partner Site Greifswald, Greifswald, Germany; 4 Department of Internal Medicine B, University Medicine Greifswald, Greifswald, Germany; 5 Department of Neurosurgery, University Medicine Greifswald, Greifswald, Germany; 6 Department of Neuroradiology, University Hospital Rostock, Rostock, Germany

**Keywords:** Idiopathic intracranial hypertension, Transverse sinus stenosis, Empty sella, Optic nerve sheath, Globe flattening, Reference range, Neurology, Brain imaging

## Abstract

Patients with chronic daily headaches (CDH) are often a diagnostic challenge and frequently undergo neuroimaging. One common underlying cause of CDH is idiopathic intracranial hypertension (IIH). However, certain neuroimaging abnormalities that suggest IIH, such as optic nerve sheath diameters (ONSD), pituitary gland height, and venous sinus diameter, require interpretation due to the absence of established normative values. Notably, intracranial pressure is known to varies with age, sex and weight, further complicating the determination of objectively abnormal findings within a specific patient group. This study aims to assist clinical neuroradiologists in differentiating neuroimaging results in CDH by providing weight-adjusted normative values for imaging characteristics of IIH. In addition to age and BMI we here assessed 1924 population-based T1-weighted MRI datasets of healthy participants for relevant MRI aspects of IIH. Association to BMI was analyzed using linear/logistic regression controlled for age and stratified for sex. ONSD was 4.3 mm [2.8; 5.9]/4.6 mm [3.6; 5.7] and diameter of transverse sinus was 4.67 mm [1.6; 6.5]/4.45 mm [3.0; 7.9]. Height of pituitary gland was 5.1 mm [2.2;8.1]/4.6 mm [1.9;7.1] for female and male respectively. Values generally varied with BMI with regression slopes spanning 0.0001 to 0.05 and were therefor presented as normative values stratified by BMI. Protrusion of ocular papilla, empty sella and transverse sinus occlusion were rare in total. Our data show an association between BMI and commonly used MRI features for diagnosing IIH. We provide categorized normative BMI values for ONSD, pituitary gland height, and transverse sinus diameter. This distinction helps objectively identify potential IIH indicators compared to regular population norms, enhancing diagnostic accuracy for suspected IIH patients. Notably, optic nerve head protrusion, empty sella, and transverse sinus occlusion are rare in healthy individuals, solidifying their importance as imaging markers regardless of BMI.

## Introduction

Chronic daily headaches (CDH) can have many different causes, which especially complicates the accurate diagnosis and thus also complicates the appropriate treatment. One common underlying cause of CDH is idiopathic intracranial hypertension (IIH). IIH first described by Quincke in 1893 as "meningitis serosa," is a disease of unknown exact pathophysiology. It most commonly affects young obese women of reproductive age. In this specific population, the incidence rate is 20 times higher than the estimated incidence of 20 per 100,000 in the general population^1–3^. Typical clinical signs include chronic daily headaches, which worsen with sudden increases in intracranial pressure such as the Valsalva maneuver. Other findings may include transient visual obscuration, retrobulbar pain, and pulsatile tinnitus, indicating increased intracranial pressure.

According to recently published evidence-based diagnostic criteria, definite IIH is still characterized by the clinical findings of papilledema and elevated cerebrospinal fluid pressure (CSF)^4^. However, neuroimaging criteria have been introduced, particularly for diagnosing IIH in instances lacking papilledema and for excluding secondary causes of elevated intracranial pressure when CSF pressure assessment is not readily accessible.

Imaging findings such as an empty sella turcica, alterations in the shape of the pituitary gland, enlarged optic nerve sheath diameter (ONSD), and stenosis or occlusion of the transverse sinus sigmoid have been reported in IIH and are included in the revised diagnostic criteria^5,6^.

This is particularly relevant since clinical findings are not specific to IIH, and imaging findings can provide valuable diagnostic information. However, there are currently no normative values for typical imaging findings in IIH, as a physiological range is expected. Additionally, body mass index (BMI) may influence these normal values, as it can physically affect cerebrospinal fluid dynamics^7^ and contribute to metabolic changes^8,9^. Therefore, establishing BMI-adjusted normative values specific to a population without headaches or neurological or ophthalmological abnormalities is crucial for accurately classifying MRI findings as abnormal and indicative of IIH.

The present study aims to address this gap by (1) examining the presence of typical imaging findings of IIH in a large population-based MRI study, (2) assessing the influence of body weight (as part of BMI), and (3) defining BMI-stratified normal values to improve the identification of pathological findings in patients with clinical suspicion of IIH.

## Material and methods

### Study population

The population-based Study of Health in Pomerania (SHIP)^10–12^ consists of two independent cohorts representing the general population: SHIP-START-0 (n = 4308) and SHIP-TREND (n = 4420), with subsequent follow-up examinations (SHIP-START-1, n = 3300 and SHIP-START-2 n = 2333)^11^. The main objective of SHIP is to obtain a representative sample of the general population to assess the incidence and prevalence of common risk factors, as well as subclinical and clinical diseases, and investigate the complex relationships between these factors and conditions. The baseline cohorts were selected from the general adult population aged 20–79 years in West Pomerania, Northeast Germany^12^. All participants gave written informed consent.

For the present analysis, the cohorts of SHIP-START-2 and SHIP-TREND-0, which provided comprehensive anamnestic, clinical, and imaging data, were utilized as the basis. Along with detailed medical history obtained through structured interviews (including headache disorders), laboratory investigations, and a comprehensive clinical examination (including neurological and ophthalmological assessments, as well as BMI assessment), a total of 3368 subjects underwent whole-body MRI (see also^12,13^).

The initial selection for further MRI evaluation included the first 2000 complete MRI datasets from participants who had no history of recurring headache within six months prior to study inclusion, no known headache disorder, no observed neurological abnormalities during clinical examination, no ophthalmological disease, no history of brain injuries or neurosurgical procedures, and no radiological abnormalities. An additional 76 participants were subsequently excluded due to low imaging quality. As a result, 1924 participants were available for the present analysis. In addition to MRI, sex, age, and BMI were recorded for all participants.

### Institutional review board statement

The studies were conducted in accordance with the Declaration of Helsinki. The study protocols were approved by the responsible local ethics committee at the University of Greifswald^12,13^.

### Cerebral MR-imaging

All T1-weighted (T1w) datasets were acquired on a 1.5T system (Magnetom Avanto; Siemens Medical Solutions, Erlangen, Germany) with the following acquisition parameters: TR = 1900 ms, TE = 3.4 ms, flip angle = 15°, spacing = 1.0 × 1.0 × 1.0 mm^3^ using a 12-channel head coil for signal detection.

### Image analysis

For image analysis, all MR datasets were transferred to an Osirix DICOM workstation (Osirix Vers. 6.0, Pixmeo, Geneva, Switzerland), and additional multiplanar reconstruction in the parasagittal and coronal planes was performed. The sagittal plane was angulated along the axis of the orbit. Image analysis was performed using a standardized window/level setting for all datasets and a standardized zoom factor of 800%. The following signs of IIH on MR images were evaluated in the T1w datasets. Despite the quality check applied to all MRI scans before their inclusion in the analysis, there were instances during the analysis where suboptimal imaging quality for specific signs, resulted in the designation of the sign as 'non-evaluable.' Consequently, it was not included in the final analysis.

#### Optic nerve diameter and optic nerve sheath diameter

**Figure 1:**
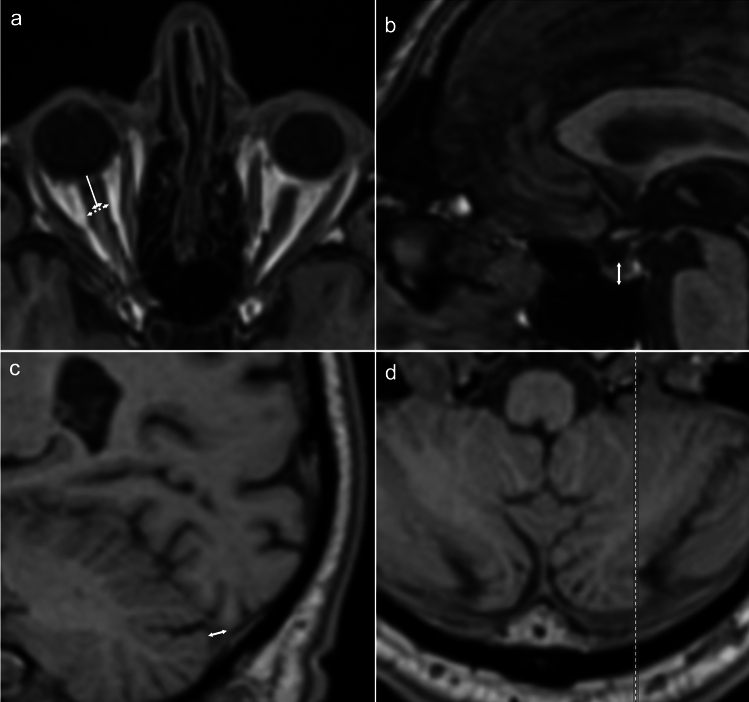
Measurement of optic nerve diameter (**a**, arrow), optic nerve sheath diameter (**a**, dotted arrow), height of the pituitary gland (**b**, arrow) and sinus transversus diameter (**c**: arrow). Middle part of sinus transversus was identified in the axial plane, measurement was than performed in axial plane (**d**: dotted line indicates sectional plane of the sagittal image **c**).

For evaluation the diameter of the optic nerve (OND) and nerve sheath (ONSD) the axial imaging plane was adjusted to perpedicular to the course of the optic nerve. The measurement was conducted separately for each side, perpendicular to the axis of the optic nerve, 8 mm posterior to the posterior circumference of the globe (Fig. 1a), as described elsewhere^14^.

#### Optic nerve tortuosity

The trajectory of each optic nerve was evaluated in the sagittal and horizontal planes using a drawn straight line. The trajectory was categorized as either straight or meandered (curved or S-shaped)^15^.

#### Shape of optic nerve head and posterior contour of the globe

In the axial plane at the level of the optic head, the shape of the globe was assessed as either flattened or round, and the optic nerve head was examined for flattening or bulging using auxiliary lines (Fig. 1a), based on^15^.

#### Height of the pituitary gland

The height of the anterior pituitary gland was measured in the median sagittal plane of the sella^16^. The individual median sagittal plane was identified for each subject (Fig. 1b). An empty sella was recorded if no identifiable anterior pituitary gland was present.

#### Diameter of the transverse sinus

The minimum diameter of the transverse sinus was measured individually for each side in the axial and sagittal planes, following the approach described by Bianchi et al.^16^ (Fig. 1c,d). If the transverse sinus was not discernible, it was categorized as hypoplastic or occluded^17,18^.

One reader (RK) examined all MRI datasets. As part of quality control, a randomly chosen subset of 70 datasets underwent re-evaluation by a board-certified neuroradiologist with 15 years of experience in brain MRI imaging. Intra-class correlation coefficients (ICC) were computed for all continuous variables, such as optic nerve sheath dimensions, pituitary gland height, and transverse sinus diameter. The ICC is a statistical method used to gauge the consistency of measurements or quantitative assessments. The ICC analysis demonstrated substantial agreement between the two raters for all continuous MRI variables (ICC > 0.9).

### Statistical analysis

Descriptive statistic of all continuous variables is provided as mean and standard of the mean. Reference values for each MR parameter are presented as median with the 2.5th and 97.5th percentiles stratified by sex, excluding cases with “empty sella” or occluded sinus transversus. Finings occurring on both sides without significant side differences were averaged for further analyses.

Association between BMI and the measured continuous MR parameters was analyzed using linear regression analysis controlled for age and stratified by sex. For categorial outcome variables (Occurrence of ……) logistic regression analyses were used.

Statistical analysis was performed with STATA version 13.1, and a p-value < 0.05 was a priori defined as indicating a statistically significant difference.

## Results

### Sample description

The final analysis comprised 1924 MRI datasets, including 950 males and 974 females. Participants had a mean age of 47.68 years (ranging from 20 to 80 years), and the mean BMI was 26.8 kg/m^2^ (ranging from 18.5 to 45.4 kg/m^2^). Further details can be found in Table 1.

**Table 1:** Descriptive statistic of all continuous variables provided as median with 2.5th and 97.5th percentiles (square bracket).

	Total	BMI groups		
		Normal [BMI < 25]	Overweight [BMI:25; < 30]	Obese [BMI≧30]
Number	1924	701	808	415
Age [yrs.]	47 [23;73]	41 [21;70]	51 [ 24;73]	52 [26; 73]
BMI [kg/m^2^]	26.5 [20.1;36.6]	22.9 [19.3;22.9]	27.2 [25.0;29.8]	32.0 [30.0;41.2]
Optic nerve diameter avg [mm]	2.4 [1.3;3.5]	2.4 [1.2;3.5]	2.4 [1.4;3.5]	2.4 [1.2;3.5]
Optic nerve sheath diameter avg [mm]	4.6 [3.5;5.8]	4.5 [3.4;5.7]	4.6 [3.5;6.0]	4.7 [3.6;5.8]
Height of the pituitary gland [mm]	4.9 [2.0;7.7]	5.1 [2.3;7.9]	4.8 [1.9;7.6]	4.8 [1.8;7.6]
Diameter of transverse sinus avg [mm]	4.4 [1.1;7.6]	3.3 [0.9;7.0]	3.2 [0.8;7.2]	3.1 [1.0;7.4]
Female	774	440	326	208
Age [yrs.]	48 [23;72]	42 [22;70]	51 [24;72]	53 [26;76]
BMI [kg/m^2^]	25.7 [19.6;38.4]	22.6 [19.0;24.8]	27.1 [25.1;29.9]	32.4 [30.0;41.2]
Optic nerve diameter avg [mm] *	2.2 [0.8;3.5]	2.4 [1.2;3.4]	2.4 [1.3;3.5]	2.5 [1.3;3.6]
Optic nerve sheath diameter avg [mm]*	4.3 [2.8;5.9]	4.5 [3.4;5.7]	4.7 [3.4;5.8]	4.7 [3.6;5.7]
Height of the pituitary gland [mm]*	5.1 [2.2;8.1]	5.4 [2.8;8.4]	5.0 [2.0;8.1]	4.8 [1.7;7.7]
Diameter of transverse sinus avg [mm]*	4,6 [1.6;6.5]	3.2 [0.9;6.9]	3.0 [0.8;6.2]	2.9 [1.0;6.1]
Male	950	260	481	209
Age [yrs.]	48 [23;75]	40 [22;70]	50 [24;75]	52 [28;77]
BMI [kg/m^2^]	27.1 [21.0;34.5]	23.5 [20.2;24.9]	27.3 [25.1;29.8]	31.8 [30.0;38.5]
Optic nerve diameter avg [mm]	2,6 [1.3;3.1]	2.4 [1.3;3.5]	2.4 [1.3;3.5]	2.4 [1.2;3.5]
Optic nerve sheath diameter avg [mm]	4.6 [3.6;5.7]	4.6 [3.4;5.9]	4.7 [3.5;6.1]	4.7 [3.7;6.0]
Height of the pituitary gland [mm]*	4.6 [1.9;7.1]	4.6 [2.1;7.1]	4.6 [1.9;7.2]	4.7 [1.9;7.0]
Diameter of transverse sinus avg [mm]*	4,4 [3.0;7.9]	3.4 [1.0;7.0]	3.4 [0.8;6.9]	3.1 [0.8.;6.8]

*Indicating significant associations to BMI.

### Presence of typical imaging findings of IIH and influence of bodyweight

#### Optic nerve diameter

**Figure 2:**
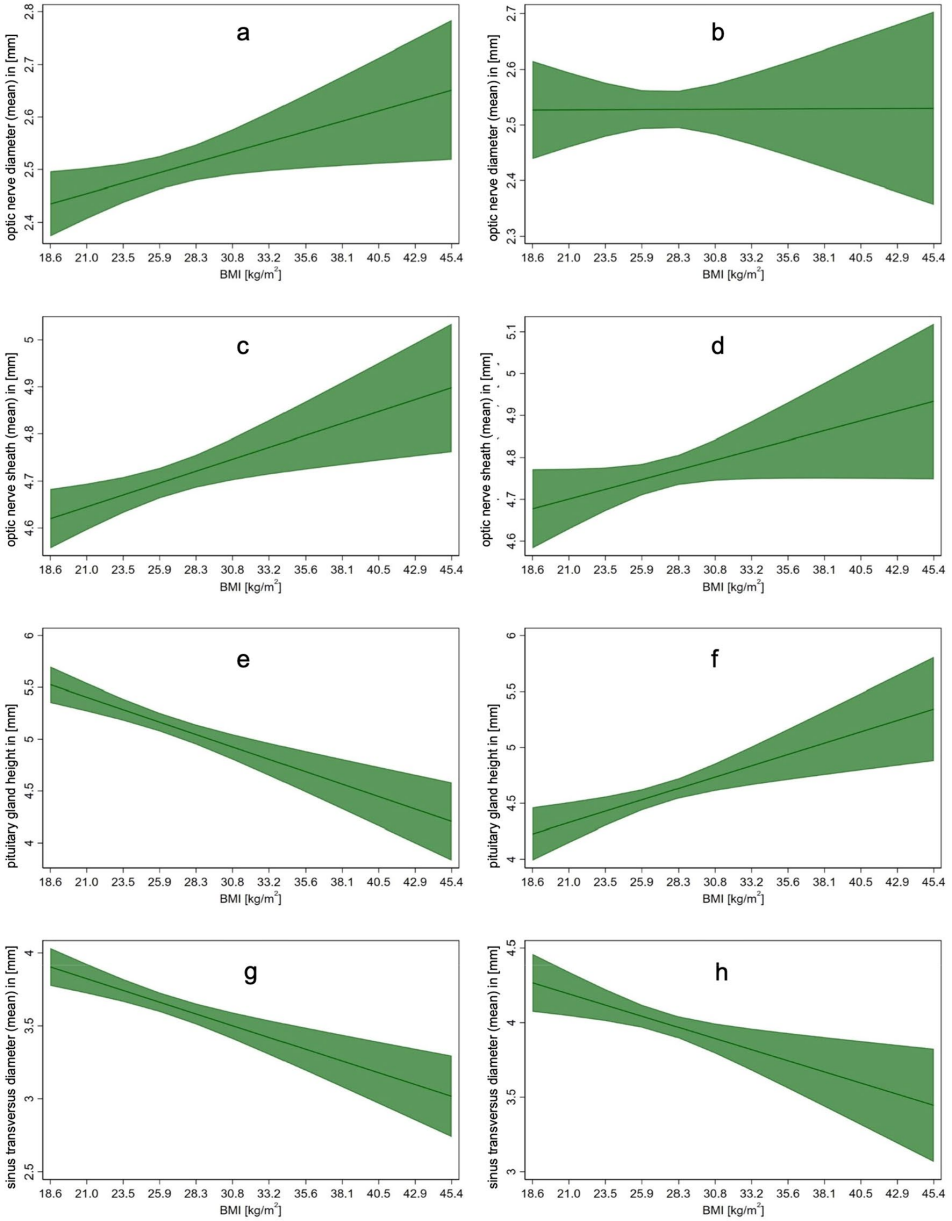
Linear regression of associations of BMI (kg/m^2^) and diameter of optic nerve (**a**,**b**), optic nerve sheath (**c**,**d**), high of pituitary gland (**e**,**f**) and sinus transversus diameter (**g**,**h**) in both female (left) and male (right).

Optic nerve diameter showed a significant correlation with BMI in women (β = 0.01, p = 0.020) but not in men (β = 0.0001, p = 0.981) (Fig. 2a,b).

#### Optic nerve sheath

For ONS, linear regression analysis reveals a significant correlation between diameter and BMI in women (β = 0.01, p = 0.004) but not in men (β = 0.01, p = 0.063) (Fig. 2c,d).

#### Optic nerve tortuosity

Logistic regression analyses showed no significant association between BMI and the likelihood of a meandered optic nerve.

#### Shape of optic nerve head and posterior contour of the globe

Logistic regression analyses did not reveal any statistically significant correlation between BMI and the likelihood of the occurrence of bulging of the optic nerve head or flattening of the posterior contour of the globe.

#### Height of the pituitary gland

There was an inverse association of BMI and height of the pituitary gland for females (β = − 0.05, p = 0.001) as opposed to males (β = 0.04, p = 0.001) (Fig. 2e,f).

#### Diameter of transverse sinus

Since body weight did not have an impact on the symmetry of the transverse sinus (absolute difference left/right: women (t (971) = − 1.28, p = 0.199; men (t (947) = 0.03, p = 0.97)), the diameters of the left and right sides were averaged for further analysis. Linear regression analysis demonstrated a significant association between BMI and sinus diameter in women (β = − 0.03, p < 0.001) and men (β = − 0.03, p = 0.004) (Fig. 2 g,h).

### Normal values stratified for body weight

Due to the significant association between body weight and most of the evaluated MRI signs, with some differences related to sex, we established normal values for the optic nerve diameter (OND), optic nerve sheath diameter (ONSD), height of the pituitary gland, and transverse sinus diameter stratified by BMI and sex.

#### Optic nerve diameter

**Figure 3:**
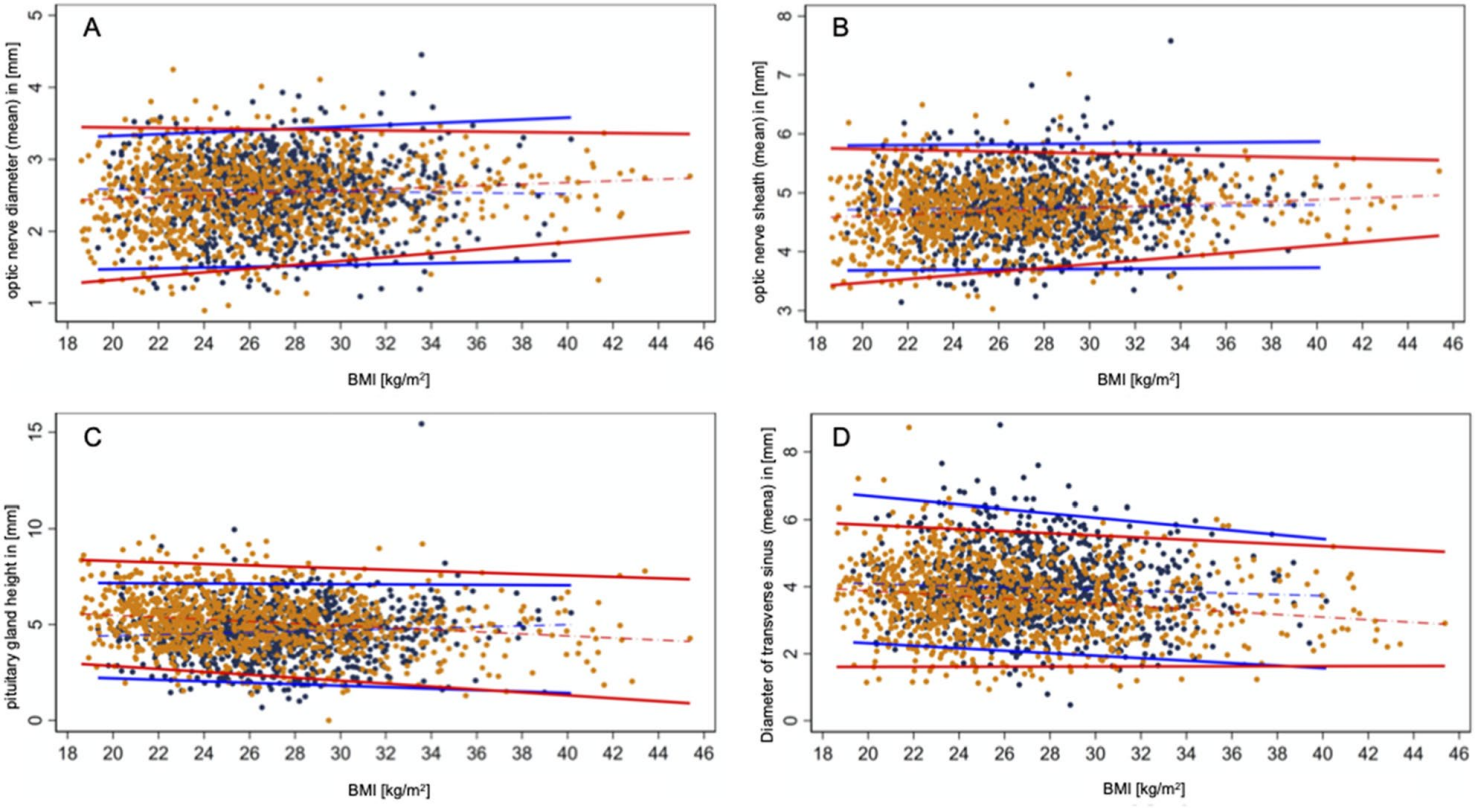
BMI-related reference ranges of optic nerve diameter (**a**), optic nerve sheath diameter (**b**), height of pituitary gland (**c**) and sinus transversus diameter (**d**) depending on BMI for both females (red lines) and males (blue lines) given as 97.5th percentile and 2.5th percentile. Dotted line indicates the median.

For the whole study population, the median of optic nerve diameter (OND) on axial T1w images was 2.2 mm [0.8;3.5] and 2,6 mm [1.3;3.1] for female and male participants respectively. (Table 1, Fig. 3a).

#### Optic nerve sheath

The median optic nerve sheath diameter (ONSD) on axial T1-weighted images was 4.3 mm [2.8; 5.9] for female participants and 4.6 mm [3.6; 5.7] for male participants in the overall study population. (Table 1, Fig. 3b).

#### Optic nerve tortuosity

**Figure 4:**
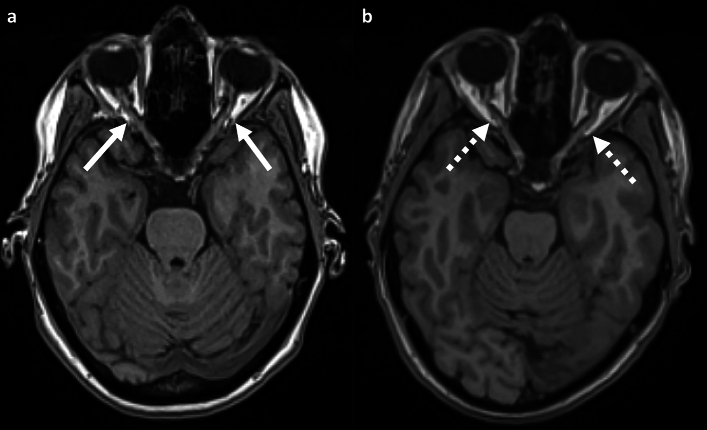
Variations in the course of the optic nerve on axial T1-weighted images; (**a**) 37-year-old female participant with straight course of the optic nerve (white arrow). (**b**) 39-year-old female participant with meandering course within the orbit (dotted white arrow).

Out of the 1924 participants, 53 individuals (2.8%; 22 females) exhibited a meandered course in one of the two optic nerves. Only one participant showed an altered course bilaterally. In 1870 participants (97.2%), the course of both optic nerves was classified as straight (Fig. 4).

#### Shape of optic nerve head and posterior contour of the globe

**Figure 5:**
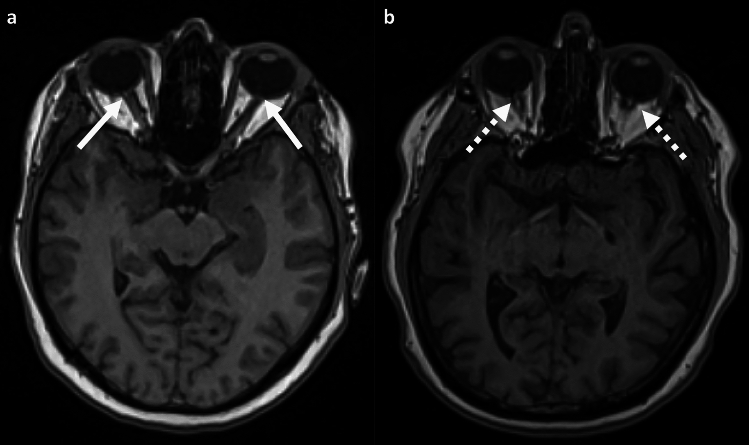
Variations in the shape of the optic disc on axial T1-weighted images. (**a**) 42-year-old male participant with flat optic disc (white arrow) and straight course of the optic nerve. (**b**) 43-year-old female participant with bulging of the optic disc (dotted white arrow), enlarged subarachnoid space and meandering course of the optic nerve.

In 1901 participants (98.8%), the ocular bulbi was classified as round, while in 19 cases (1%; 4 females), both bulbi were flattened. Only 4 participants (0.2%, 2 females) showed unilateral flattening of the ocular bulbus. The ocular papilla was flat in 1878 participants (97.6%), but in 46 participants (2.4%, 20 females), the ocular papilla showed a significant protrusion. This protrusion was observed bilaterally in 6 cases and unilaterally in 40 cases (Fig. 5).

#### Height of the pituitary gland

Median height of pituitary gland in sagittal T1w images was 5.1 [2.2;8.1] and 4.6 [1.9;7.1] for female and male respectively. (Table 1, Fig. 3c).

**Figure 6:**
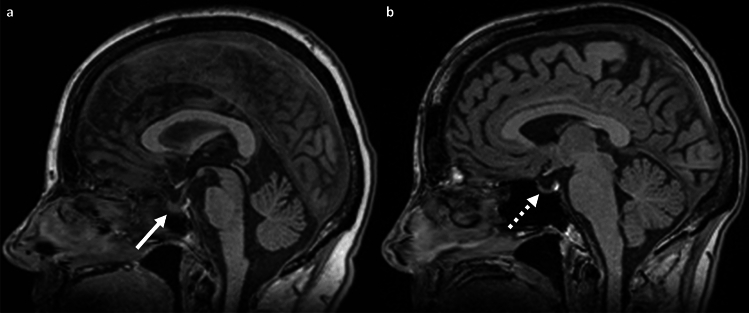
Variations of the pituitary gland on midline sagittal reconstructed T1-weighted images; (**a**) 29-year-old male participant (BMI 25.4 kg/m^2^) with normal height of the pituitary gland (white arrow). (**b**) 32-year-old male participant with empty sella phenomenon (dotted white arrow).

In 3.3% (n = 64), an “empty sella” phenomenon was observed without significant difference in sex. (Fig. 6).

#### Diameter of transverse sinus

**Figure 7:**
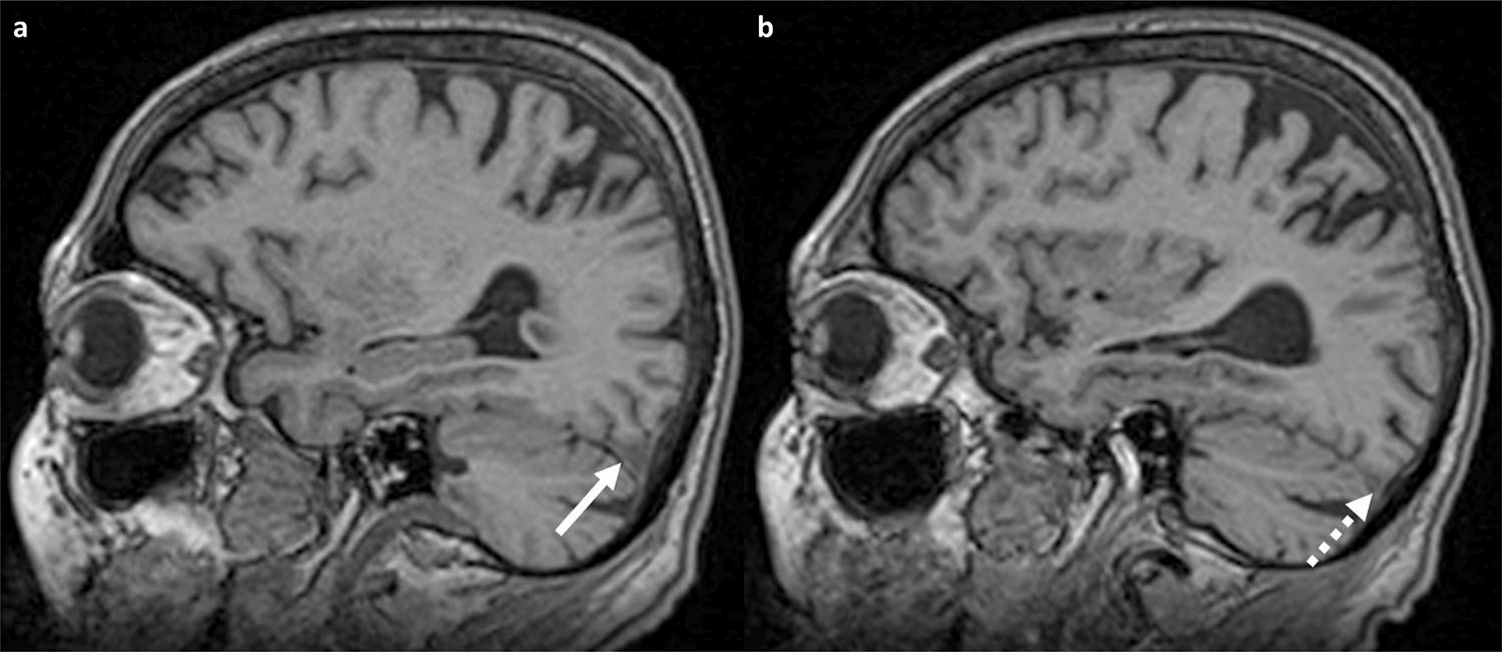
Variations of the transverse sinus on parasagittal reconstructed T1-weighted images; (**a**) 78-year-old female patient with normal appearing transverse sinus (white arrow). (**b**) 80-year-old female patient with non-detectable transverse sinus (dotted white arrow) at the identical slice position rated as occluded/stenotic transverse sinus.

The mean diameter of the transverse sinus on axial T1-weighted images was 4.67 mm [1.6; 6.5] for females and 4.45 mm [3.0; 7.9] for males (Table 1, Fig. 3d). In 57 participants (3%, 28 females), at least one of the transverse sinuses (right: n = 24; left: n = 29; both: n = 4) was not detectable on axial and sagittal images and was therefore classified as occluded or absent. No significant difference was observed based on sex (Fig. 7).

## Discussion

Imaging plays a crucial role in the diagnostic work-up of patients with suspected IIH by aiding in the exclusion of secondary causes. In our population-based MRI study, we observed a significant association between BMI and several important imaging parameters, including OND, ONSD, height of the pituitary gland, and transverse sinus diameter. These findings are particularly relevant as they are considered key imaging features in the diagnosis of IIH.

### Optic nerve diameter, optic nerve sheath diameter and course of the optic nerve

The mean values of optic nerve diameter in our study align well with existing literature on healthy participants^17^. Although there were no significant differences in the mean values based on sex, we observed a significant association between optic nerve diameter and BMI, specifically in women. However, the medical significance of OND in the pathophysiology of neurological diseases remains unclear, as MRI studies focusing on this parameter are rare.

In contrast, the enlargement of ONSD is directly associated with intracranial pressure, both in healthy individuals^18^ and in patients with IIH^19^.

Although sensitivity (0.45–0.66) and specificity (0.82–0.95) of enlarged ONSD in relation to elevated intracranial pressure vary widely in the literature^20^, this MRI parameter is frequently utilized in the diagnostic process. On MRI, enlargement of the optic nerve sheath (ONS) appears as a widened ring of cerebrospinal fluid (CSF) around the intraorbital course of the optic nerve with isointense signal to CSF on both sides of the nerve in axial and coronal images. The ONSD in our study is comparable to previous reports^14^. Unlike previous ultrasound studies, we did not observe any differences between men and women in our MRI measurements^20^, but we did find a positive correlation between ONSD and BMI, which has not been previously described^21^.

Interestingly, a meandering course of the optic nerve, optic nerve head protrusion, and flattening of the posterior aspect of the globe were observed in less than 2% of all participants. We therefore postulate that ONSD is a highly sensitive surrogate parameter for intracranial pressure, which may be increased due to higher BMI without being clinically relevant^22^.

Conversely, findings like meandering course of the optic nerve, optic nerve head protrusion, and flattening of the globe might occur more frequently in patients with clinically relevant increased intracranial pressure. This conclusion is supported by a recent study by Witsberger et al.^23^ in which they suggest that raised intracranial pressure is a continuum, with even asymptomatic individuals demonstrating abnormal findings on brain MRI. Our assumptions are further supported by a recent meta-analysis of IIH patients by Kwee and colleagues^6^ who reported high specificity for optic nerve course, optic nerve head protrusion, and flattening of the posterior globe in patients with IIH, while ONS diameter exhibited high variability in the analyzed literature.

### Height of the pituitary gland

Sex-related differences in hormonal activity are thought to be responsible for the observed differences in pituitary gland height, with larger pituitary glands observed in females^24^. In female patients, we also identified a correlation between pituitary gland size and BMI. These findings align with a recent study by Witsberger et al.^23^, who observed a direct correlation between the ratio of pituitary gland height relative to the sella and opening pressure on lumbar puncture for CSF analysis. However, we observed inverse association of BMI and height of the of pituitary gland for females as opposed to males. Whether this observation is related to differences in hormonal activity needs to be further evaluated in subgroup analyses. The presence of an empty sella was observed in 3.3% of our study population, which is lower than the prevalence reported in previous studies^25^. We did not find any correlation between BMI and the presence of an empty sella, regardless of sex. In the interpretation of the findings, it is essential to emphasize that we are presenting the height of the pituitary rather than its ratio to the sella^4^. Therefore, a precise categorization of pituitary morphology based on the degree of suprasellar herniation of cerebrospinal fluid into the sella, as conducted by other authors, is not feasible.

### Diameter of the transverse sinus

In a recent meta-analysis by Kwee et al., it was reported that alterations in the diameter of the transverse sinus were the only MRI sign with high pooled specificity and reasonably high sensitivity in diagnosing IIH^6^. Previous studies have also reported a negative correlation between invasively measured venous pressure gradients in the transverse sinus and the diameter of the sinus in patients with IIH^26^. However, our data demonstrates a significant negative correlation between sinus diameter and BMI. This finding may support the hypothesis of a relationship between overweight, increased intraabdominal pressure, subsequent extracranial venous pressure, and increased intracranial pressure, resulting in a decreased diameter of the sinus. Whereas the rate of transverse sinus occlusion in patients with secondary IIH can be as high as 94%^27^ we observed transverse sinus occlusion in our healthy cohort on one side only in 2.7% of participants and bilaterally in 0.2%. These findings are consistent with previous studies^28^ and emphasize the significance of the radiological finding of sinus occlusion in the diagnostic process of IIH.

The present study has several strengths, including its large sample size derived from a representative general population. The retrospective design allowed for the assessment of the effects of sex and BMI on the evaluated MRI parameters. However, there are also limitations to consider. Firstly, it should be noted that the analysis was exclusively conducted using T1 images, which often is a common practice in clinical settings. However, the significance of the findings could potentially be enhanced by incorporating additional 3D heavily T2-weighted images and dedicated MR venography sequences. The absence of the latter limited the evaluation of the transverse sinus especially since diameter measurement was performed only at a predefined position rather than along the entire course of the sinus.

Secondly, the absence of data regarding lumbar puncture opening pressure, a pivotal method in the diagnostic evaluation of suspected idiopathic intracranial hypertension (IIH), poses a limitation. This is particularly noteworthy given the apparent association between adipose tissue and intracranial hypertension. However, the precise correlation with the current cerebrospinal fluid (CSF) pressure remains unclear. The actual pressure seems to be more closely associated with CSF production and absorption^29^. Conversely, BMI appears to play a role in the predisposition and likelihood of developing the condition. Based on our data, one can only speculate about the potential influence of BMI on the opening pressure itself. Notably, the European Headache Federation (EHF) Guidelines recognize a grey zone between 25–30 cm H2O in obese patients. It may be appropriate to state that the influence of BMI on the opening pressure is "uncertain," but referring to the EHF guidelines aligns well with our findings, suggesting that the opening pressure might be "marginally" affected^30^.

Additionally, due to the representative nature of the data for the general population, there were only a few cases of suspected or diagnosed IIH included, which limited the availability of a direct patient control group. Future studies should aim to replicate the reference data obtained from healthy individuals in a large cohort of patients with IIH to further validate the findings.

## Conclusion

In a population-based MRI study involving participants without any clinical evidence of headache disease, we identified significant correlations between BMI and parameters such as ONSD, pituitary gland height, and transverse sinus diameter. These correlations hold implications for the diagnostic evaluation of patients with suspected IIH. Our study contributes BMI-stratified normal values that complement the existing literature and can enhance diagnostic precision in the future.

Furthermore, we observed that specific imaging criteria, such as the course of the optic nerve, protrusion of the optic nerve head, and occlusion of the transverse sinus, were infrequent among asymptomatic individuals. Despite considering BMI, these criteria could have significance in the diagnostic process for patients suspected of IIH.

Aligning with our findings, recent literature has shown high sensitivity and notably exceptional specificity of various MRI aspects, but only when employed as a composite diagnostic criterion for IIH^4,31^. Nevertheless, this underscores their potential significance in the clinical assessment of suspected IIH.

### Supplementary Information

Supplementary Information.

## Data Availability

Data are subject to national data protection laws and restrictions were imposed by the Medical Ethics Committee of the University Medicine of Greifswald. The informed consent signed by the SHIP study subjects includes the statement that the data will not be made available as a public use file. Data are however available upon formal request. Instruction for application concerning delivery and use of data and/or sample material are provided under https://www.fvcm.med.uni-greifswald.de. Those interested can access the data in the same manner as the authors. The authors had no special access privileges to the data. For any questions regarding the application process and data handling the researcher can contact transfer@uni-greifswald.de.

## Abbreviations

BMI: Body Mass Index
CSF: Cerebrospinal fluid
ICC: Intra-class correlation coefficients
IIH: Idiopathic intracranial hypertension
ON: Optic nerve
OND: Diameter of the optic nerve
ONSD: Optic nerve sheath diameter
SHIP: Study of Health in Pomerania

## References

[1] Jensen RH, Radojicic A, Yri H (2016). The diagnosis and management of idiopathic intracranial hypertension and the associated headache. Ther. Adv. Neurol. Disord..

[2] Durcan FJ, Corbett JJ, Wall M (1988). The incidence of pseudotumor cerebri. Population studies in Iowa and Louisiana. Arch Neurol.

[3] Radhakrishnan K, Ahlskog JE, Cross SA, Kurland LT, O’Fallon WM (1993). Idiopathic intracranial hypertension (pseudotumor cerebri). Descriptive epidemiology in Rochester, Minn, 1976 to 1990. Arch. Neurol..

[4] Korsbæk JJ, Jensen RH, Høgedal L, Molander LD, Hagen SM, Beier D (2023). Diagnosis of idiopathic intracranial hypertension: A proposal for evidence-based diagnostic criteria. Cephalalgia.

[5] Brodsky MC, Vaphiades M (1998). Magnetic resonance imaging in pseudotumor cerebri. Ophthalmology.

[6] Kwee RM, Kwee TC (2019). Systematic review and meta-analysis of MRI signs for diagnosis of idiopathic intracranial hypertension. Eur. J. Radiol..

[7] Wakerley BR, Warner R, Cole M, Stone K, Foy C, Sittampalam M (2020). Cerebrospinal fluid opening pressure: The effect of body mass index and body composition. Clin. Neurol. Neurosurg..

[8] Berdahl JP, Fleischman D, Zaydlarova J, Stinnett S, Allingham RR, Fautsch MP (2012). Body mass index has a linear relationship with cerebrospinal fluid pressure. Investig. Ophthalmol. Vis. Sci..

[9] Westgate CSJ, Israelsen IME, Jensen RH, Eftekhari S (2021). Understanding the link between obesity and headache: With focus on migraine and idiopathic intracranial hypertension. J. Headache Pain.

[10] Volzke H, Alte D, Schmidt CO, Radke D, Lorbeer R, Friedrich N, Aumann N, Lau K, Piontek M, Born G, Havemann C, Ittermann T, Schipf S, Haring R, Baumeister SE, Wallaschofski H, Nauck M, Frick S, Arnold A, Junger M, Mayerle J, Kraft M, Lerch MM, Dorr M, Reffelmann T, Empen K, Felix SB, Obst A, Koch B, Glaser S, Ewert R, Fietze I, Penzel T, Doren M, Rathmann W, Haerting J, Hannemann M, Ropcke J, Schminke U, Jurgens C, Tost F, Rettig R, Kors JA, Ungerer S, Hegenscheid K, Kuhn JP, Kuhn J, Hosten N, Puls R, Henke J, Gloger O, Teumer A, Homuth G, Volker U, Schwahn C, Holtfreter B, Polzer I, Kohlmann T, Grabe HJ, Rosskopf D, Kroemer HK, Kocher T, Biffar R, John U, Hoffmann W (2011). Cohort profile: The study of health in Pomerania. Int. J. Epidemiol..

[11] Völzke H, Schössow J, Schmidt CO, Jürgens C, Richter A, Werner A, Werner N, Radke D, Teumer A, Ittermann T, Schauer B, Henck V, Friedrich N, Hannemann A, Winter T, Nauck M, Dörr M, Bahls M, Felix SB, Stubbe B, Ewert R, Frost F, Lerch MM, Grabe HJ, Bülow R, Otto M, Hosten N, Rathmann W, Schminke U, Großjohann R, Tost F, Homuth G, Völker U, Weiss S, Holtfreter S, Bröker BM, Zimmermann K, Kaderali L, Winnefeld M, Kristof B, Berger K, Samietz S, Schwahn C, Holtfreter B, Biffar R, Kindler S, Wittfeld K, Hoffmann W, Kocher T (2022). Cohort profile update: The Study of Health in Pomerania (SHIP). Int. J. Epidemiol..

[12] Völzke H, Alte D, Schmidt CO, Radke D, Lorbeer R, Friedrich N, Aumann N, Lau K, Piontek M, Born G, Havemann C, Ittermann T, Schipf S, Haring R, Baumeister SE, Wallaschofski H, Nauck M, Frick S, Arnold A, Jünger M, Mayerle J, Kraft M, Lerch MM, Dörr M, Reffelmann T, Empen K, Felix SB, Obst A, Koch B, Gläser S, Ewert R, Fietze I, Penzel T, Dören M, Rathmann W, Haerting J, Hannemann M, Röpcke J, Schminke U, Jürgens C, Tost F, Rettig R, Kors JA, Ungerer S, Hegenscheid K, Kühn JP, Kühn J, Hosten N, Puls R, Henke J, Gloger O, Teumer A, Homuth G, Völker U, Schwahn C, Holtfreter B, Polzer I, Kohlmann T, Grabe HJ, Rosskopf D, Kroemer HK, Kocher T, Biffar R, John U, Hoffmann W (2011). Cohort profile: The study of health in Pomerania. Int. J. Epidemiol..

[13] John U, Greiner B, Hensel E, Lüdemann J, Piek M, Sauer S, Adam C, Born G, Alte D, Greiser E, Haertel U, Hense HW, Haerting J, Willich S, Kessler C (2001). Study of Health in Pomerania (SHIP): A health examination survey in an east German region: Objectives and design. Soz Praventivmed..

[14] Vaiman M, Abuita R, Bekerman I (2015). Optic nerve sheath diameters in healthy adults measured by computer tomography. Int. J. Ophthalmol..

[15] Agid R, Farb RI, Willinsky RA, Mikulis DJ, Tomlinson G (2006). Idiopathic intracranial hypertension: The validity of cross-sectional neuroimaging signs. Neuroradiology.

[16] Lamichhane TR, Pangeni S, Paudel S, Lamichhane HP (2015). Age and gender related variations of pituitary gland size of healthy nepalese people using magnetic resonance imaging. Am. J. Biomed. Eng..

[17] Mncube SS, Goodier MD (2019). Normal measurements of the optic nerve, optic nerve sheath and optic chiasm in the adult population. SA J. Radiol..

[18] Passi N, Degnan AJ, Levy LM (2013). MR imaging of papilledema and visual pathways: Effects of increased intracranial pressure and pathophysiologic mechanisms. AJNR Am. J. Neuroradiol..

[19] Gass A, Barker GJ, Riordan-Eva P, MacManus D, Sanders M, Tofts PS, McDonald WI, Moseley IF, Miller DH (1996). MRI of the optic nerve in benign intracranial hypertension. Neuroradiology.

[20] Goeres P, Zeiler FA, Unger B, Karakitsos D, Gillman LM (2016). Ultrasound assessment of optic nerve sheath diameter in healthy volunteers. J. Crit. Care.

[21] Rohr A, Riedel C, Reimann G, Alfke K, Hedderich J, Jansen O (2008). Pseudotumor cerebri: Quantitative in-vivo measurements of markers of intracranial hypertension. Rofo.

[22] Kilic K, Korsbæk JJ, Jensen RH, Cvetkovic VV (2022). Diagnosis of idiopathic intracranial hypertension—the importance of excluding secondary causes: A systematic review. Cephalalgia.

[23] Witsberger EM, Huston J, Cutsforth-Gregory JK, Johnson PW, Bhatti MT, Chen JJ (2022). Population-based evaluation of indirect signs of increased intracranial pressure. J. Neuroophthalmol..

[24] Tsunoda A, Okuda O, Sato K (1997). MR height of the pituitary gland as a function of age and sex: Especially physiological hypertrophy in adolescence and in climacterium. AJNR Am. J. Neuroradiol..

[25] De Marinis M, Pujia A, Natale L, D'Arcangelo E, Accornero N (2003). Decreased habituation of the R2 component of the blink reflex in migraine patients. Clin. Neurophysiol..

[26] West JL, Greeneway GP, Garner RM, Aschenbrenner CA, Singh J, Wolfe SQ, Fargen KM (2019). Correlation between angiographic stenosis and physiologic venous sinus outflow obstruction in idiopathic intracranial hypertension. J. Neurointerv. Surg..

[27] Morris PP, Black DF, Port J, Campeau N (2017). Transverse sinus stenosis is the most sensitive MR imaging correlate of idiopathic intracranial hypertension. AJNR Am. J. Neuroradiol..

[28] Kelly LP, Saindane AM, Bruce BB, Ridha MA, Riggeal BD, Newman NJ, Biousse V (2013). Does bilateral transverse cerebral venous sinus stenosis exist in patients without increased intracranial pressure?. Clin. Neurol. Neurosurg..

[29] Mitchell JL, Lyons HS, Walker JK, Yiangou A, Grech O, Alimajstorovic Z, Greig NH, Li Y, Tsermoulas G, Brock K, Mollan SP, Sinclair AJ (2023). The effect of GLP-1RA exenatide on idiopathic intracranial hypertension: A randomized clinical trial. Brain.

[30] Hoffmann J, Mollan SP, Paemeleire K, Lampl C, Jensen RH, Sinclair AJ (2018). European Headache Federation guideline on idiopathic intracranial hypertension. J. Headache Pain.

[31] Friedman DI, Liu GT, Digre KB (2013). Revised diagnostic criteria for the pseudotumor cerebri syndrome in adults and children. Neurology.

