# Supplementary material for: Normative body mass-adjusted reference ranges of magnetic resonance imaging signs commonly used in diagnosing idiopathic intracranial hypertension in a healthy standard population
Source: Sci Rep. 2024 Feb 24;14:4492. doi: 10.1038/s41598-024-54975-0 (PMC10891171; doi:10.1038/s41598-024-54975-0)
Supplement: Supplementary file 1 — Supplementary Information. [file 41598_2024_54975_MOESM1_ESM.docx]

**Supplementary material**

Normative body mass-adjusted reference ranges of magnetic resonance imaging signs commonly used in diagnosing idiopathic intracranial hypertension in a healthy standard population

***Authors***

Rike Kobrow^1^, Stefan Gross^3,4^ , Robert Fleischmann^2^, Jörg Baldauf^5^, Sönke Langner^6^, Sebastian Strauss^2^

*1 Institute of Diagnostic Radiology and Neuroradiology, University Medicine Greifswald, Greifswald, Germany.*

*2 Department of Neurology, University Medicine Greifswald, Greifswald, Germany*

*3 DZHK (German Center for Cardiovascular Research), Partner Site Greifswald, Greifswald, Germany.*

*4 Department of Internal Medicine B, University Medicine Greifswald, Greifswald, Germany.*

*5 Department of Neurosurgery, University Medicine Greifswald, Greifswald, Germany.*

*6 Department of Neuroradiology, University Hospital Rostock, Germany*

***Corresponding Author***

Dr. med. Sebastian Strauss, MD

Department of Neurology, University Medicine Greifswald

Ferdinand-Sauerbruch-Str. 1, 17475 Greifswald, Germany

Phone: +49-3834-86-6855

Email: [sebastian.strauss@med.uni-greifswald.de](mailto:sebastian.strauss@med.uni-greifswald.de)

**Supplementary results**

Supplementary Figure 1: BMI-related reference ranges of optic nerve diameter (a;b), optic nerve sheath diameter (c;d), height of pituitary gland (e;f) and sinus transversus diameter (g;h) depending on BMI for both females and males given as 97.5^th^ percentile and 2.5^th^ percentile separately for different age groups. Dotted line indicates the median.

**
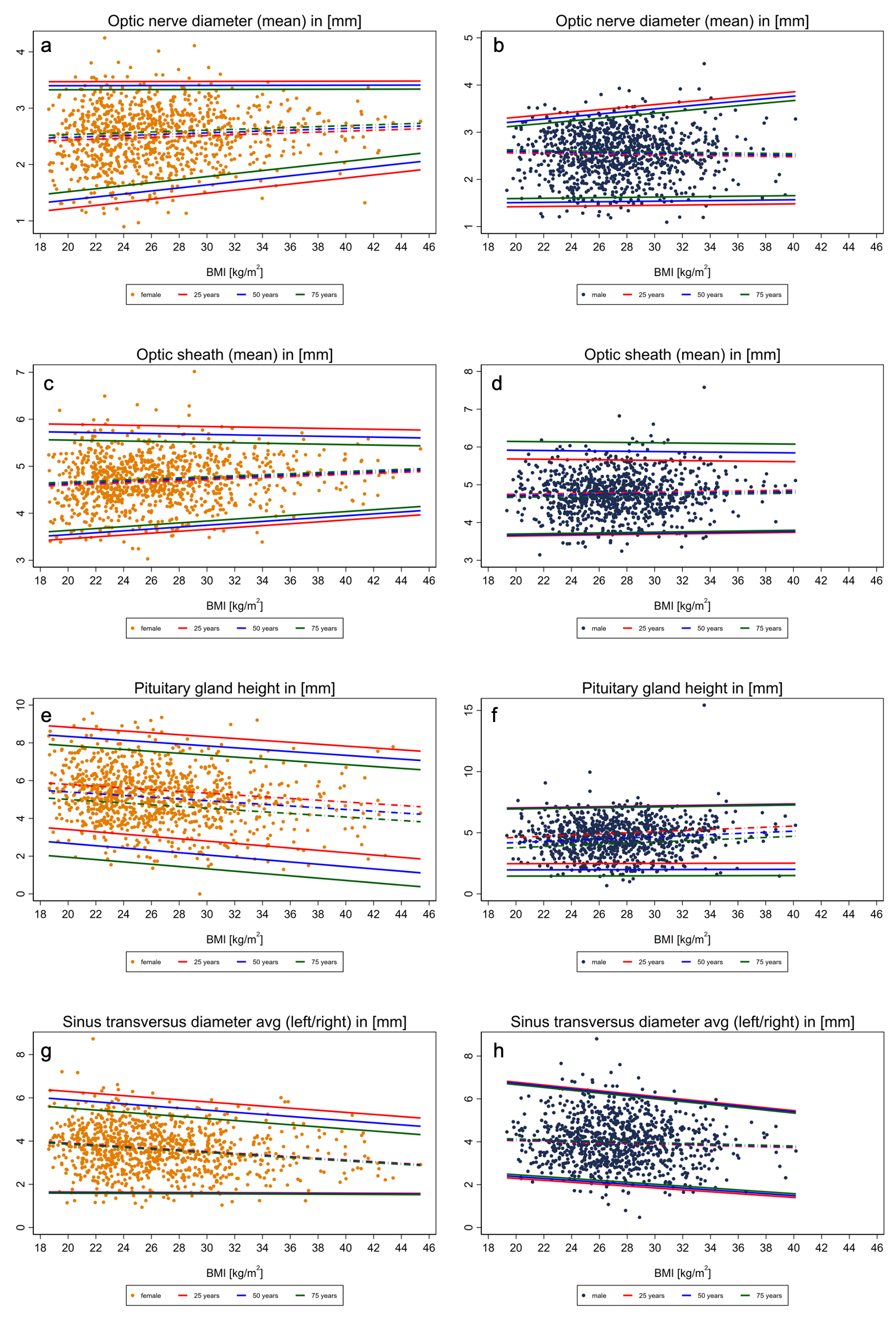
**
